# FGF‐2 promotes osteocyte differentiation through increased E11/podoplanin expression

**DOI:** 10.1002/jcp.26345

**Published:** 2018-01-23

**Authors:** Ekele Ikpegbu, Lena Basta, Dylan N. Clements, Robert Fleming, Tonia L. Vincent, David J. Buttle, Andrew A. Pitsillides, Katherine A. Staines, Colin Farquharson

**Affiliations:** ^1^ Roslin Institute and R(D)SVS The University of Edinburgh Edinburgh UK; ^2^ Michael Okpara University of Agriculture Abia Nigeria; ^3^ Arthritis Research UK Centre for Osteoarthritis Pathogenesis, Kennedy Institute of Rheumatology University of Oxford Oxford UK; ^4^ Department of Infection, Immunity & Cardiovascular Disease The University of Sheffield Medical School Sheffield UK; ^5^ Comparative Biomedical Sciences The Royal Veterinary College London UK; ^6^ School of Applied Sciences Edinburgh Napier University Edinburgh UK

**Keywords:** E11/podoplanin, FGF‐2, osteoblasts, osteocytes, osteocytogenesis

## Abstract

E11/podoplanin is critical in the early stages of osteoblast‐to‐osteocyte transitions (osteocytogenesis), however, the upstream events which regulate E11 expression are unknown. The aim of this study was to examine the effects of FGF‐2 on E11‐mediated osteocytogenesis and to reveal the nature of the underlying signaling pathways regulating this process. Exposure of MC3T3 osteoblast‐like cells and murine primary osteoblasts to FGF‐2 (10 ng/ml) increased *E11* mRNA and protein expression (*p* < 0.05) after 4, 6, and 24 hr. FGF‐2 induced changes in E11 expression were also accompanied by significant (*p* < 0.01) increases in *Phex* and *Dmp1* (osteocyte markers) expression and decreases in *Col1a1*, *Postn*, *Bglap*, and *Alpl* (osteoblast markers) expression. Immunofluorescent microscopy revealed that FGF‐2 stimulated E11 expression, facilitated the translocation of E11 toward the cell membrane, and subsequently promoted the formation of osteocyte‐like dendrites in MC3T3 and primary osteoblasts. siRNA knock down of E11 expression achieved >70% reduction of basal *E11* mRNA expression (*p* < 0.05) and effectively abrogated FGF‐2‐related changes in E11 expression and dendrite formation. FGF‐2 strongly activated the ERK signaling pathway in osteoblast‐like cells but inhibition of this pathway did not block the ability of FGF‐2 to enhance E11 expression or to promote acquisition of the osteocyte phenotype. The results of this study highlight a novel mechanism by which FGF‐2 can regulate osteoblast differentiation and osteocyte formation. Specifically, the data suggests that FGF‐2 promotes osteocytogenesis through increased E11 expression and further studies will identify if this regulatory pathway is essential for bone development and maintenance in health and disease.

## 1 INTRODUCTION

Osteocytes are derived from osteoblasts and are the most abundant cells, residing in mineralized bone of the adult skeleton. It has long been accepted that osteocytes are formed by the passive entrapment of redundant osteoblasts by osteoid synthesized by their close neighbors (Palumbo, Ferretti, & Marotti, 2004; Skerry, Bitensky, Chayen, & Lanyon, 1989). The transition from the cuboidal‐like osteoblastic morphology to a dendritic shape characteristic of an osteocyte is, however, a more active and tightly regulated process than originally recognized (for reviews see Dallas & Bonewald, 2010; Franz‐Odendaal, Hall, & Witten, 2006).

The mechanisms which govern this osteoblast to osteocyte transition (osteocytogenesis) are generally unknown but fundamental studies by Bonewald and coworkers identified E11/podoplanin, a mucin‐type transmembrane glycoprotein, as the earliest osteocyte marker protein expressed during osteocytogenesis (Zhang et al., 2006). Furthermore, E11 triggers actin cytoskeletal dynamics (Staines et al., 2016), which are required for dendrite formation and transient *E11* knockdown blocks dendrite elongation (Zhang et al., 2006). E11 glycoprotein is not unique to bone and is ubiquitously expressed by many tissues in which it has a range of regulatory functions including cell development, differentiation and invasiveness, epithelial–mesenchymal transition, and oncogenesis (Astarita, Acton, & Turley, 2012; Martín‐Villar, Yurrita, Fernández‐Muñoz, Quintanilla, & Renart, 2009; Thiery, 2002; Wicki & Christofori, 2007). Owing to its wide tissue expression, it is now recognized by several names which include podoplanin in kidney podocytes, T1α in alveolar type 1 epithelial cells, PA2.26 in skin keratinocytes, gp38 in lymphoid organs, and E11 in lymphatic endothelial cells, osteoblasts, and osteocytes (Breiteneder‐Geleff et al., 1997; Farr, Nelson, & Hosier, 1992; Ramirez et al., 2003; Scholl, Gamallo, Vilaró, & Quintanilla, 1999; Wetterwald et al., 1996).

The intracellular signaling mechanisms by which E11 influences dendrite formation involve the activation of the small GTPase, RhoA, and its downstream effector kinase, ROCK (Martín‐Villar et al., 2006). ROCK phosphorylates ezrin/moesin/radixin (ERM) and influences the actin cytoskeleton and subsequently cell shape (Martín‐Villar et al., 2014, 2006; Sprague, Wetterwald, Heinzman, & Atkinson, 1996). Much less, however, is known about the upstream regulatory events, specifically those that influence levels of E11 expression during osteocytogenesis. Nonetheless, clues from other model systems have indicated that fibroblast growth factor 2 (FGF‐2) is able to change chondrocyte gene expression in vitro, including that of *E11* (Chong et al., 2013). FGF‐2, one of the earliest members identified in the FGF polypeptide family, signals through FGF receptors that have intrinsic tyrosine kinase activity (Powers, Mcleskey, & Wellstein, 2000). In addition to chondrocytes, FGF‐2 is expressed by osteoblasts and is stored in the extracellular matrix where it regulates bone formation via influence on progenitor cell lineage commitment and/or osteoblast differentiation (Hurley, Marie, & Florkiewicz, 2002; Montero et al., 2000; Sabbieti et al., 1999; Xiao et al., 2010). Indeed, mice deficient in *Fgf2* have decreased bone mass and altered trabecular architecture whereas *Fgf2* transgenic mice present with increased bone mineral density and cortical and trabecular thickness, as well as a variety of skeletal malformations including shortening and flattening of long bones (Coffin et al., 1995; Montero et al., 2000; Xiao et al., 2009).

Cognizant of FGF‐2 stimulation of E11 expression in cartilage explants and osteoblast‐like cells, we, therefore, hypothesized that FGF‐2 may influence bone remodeling via increased osteoblast E11 expression and concomitant osteocyte dendrite formation (Chong et al., 2013; Gupta, Yoo, Hebert, Niger, & Stains, 2010). Hence, the aims of this current study were to examine the effects of FGF‐2 on E11 expression in osteoblasts during osteocytogenesis and to explore putative signaling pathways controlling this process.

## 2 MATERIALS AND METHODS

### 2.1 Animals

FGF‐2‐deficient mice (KO) were originally created by Tom Doetschman and obtained from the Jackson Laboratory, and were backcrossed onto a C57BL/6J wild‐type (WT) background (Chong et al., 2013). Animal experiments were performed after obtaining ethical and statutory approval in accordance with local policy. Mice were maintained in accordance with UK Home Office guidelines for the care and use of laboratory animals.

### 2.2 MC3T3 cell culture

Murine MC3T3‐E1 (subclone 14), pre‐osteoblast‐like cells (American Type Culture Collection [ATCC], Manassas, VA) were plated at 1 × 10^4^ cells/cm^2^ in six‐well plates and cultured in α‐MEM medium supplemented with 10% (v/v) FBS (Invitrogen, Paisley UK) and 50 µg/ml gentamicin (Invitrogen) at 37°C in a humidified atmosphere with 5% CO_2_ and the medium was changed every 2–3 days. Cell viability was assessed using a commercially available Alamar Blue kit (Invitrogen) and cell cytotoxicity using an LDH assay according to the manufacturer's instructions (Promega, Southampton, UK).

### 2.3 Primary osteoblast isolation

Primary calvarial osteoblasts were obtained from 3‐day‐old WT mice by serial enzyme digestion of dissected calvarial bones according to published procedure (Orriss, Hajjawi, Huesa, Macrae, & Arnett, 2014; Staines, Zhu, Farquharson, & Macrae, 2014). In brief, calvaria were digested in 1 mg/ml collagenase type II (Thermo Fisher Scientific, Loughborough, UK) in Hanks’ balanced salt solution (HBSS) for 10 min and the supernatant discarded; then repeat digestion in 1 mg/ml collagenase type II in HBSS for 30 min; 4 mM EDTA for 10 min and finally 1 mg/ml collagenase type II in HBSS for 30 min. After discarding the first digest, the cells were re‐suspended in growth medium consisting of α‐MEM supplemented with 10% (v/v) FBS and gentamycin at 50 μg/ml. Osteoblasts were seeded at a density of 1 × 10^4^ cells/cm^2^, and incubated at 37°C/5%CO_2_ with media changes every 2–3 days.

### 2.4 FGF‐2 treatments

When MC3T3 cells and primary osteoblasts were confluent (day 0), the culture media were replaced with α‐MEM supplemented with 1% (v/v) FBS, 50 µg/ml gentamicin and 0‐50 ng/ml FGF‐2 (PeproTech, London, UK) in 0.1% bovine serum albumin (BSA). Each test condition was completed in triplicate.

### 2.5 Signaling inhibitors

MC3T3 cells were incubated with appropriate concentrations (specific details in results) of the MEK1/2 inhibitor, U0126, the PI3K inhibitor, LY294002 (InvivoGen, Toulouse, France), and the p38 inhibitor, SB203580 (Cell Guidance Systems, Cambridge, UK). These inhibitors have been reported to be selective for these molecules (Choi et al., 2008; Hotokezaka et al., 2002; Macrae, Ahmed, Mushtaq, & Farquharson, 2007). Control cultures contained vehicle (0.1% dimethylsulfoxide, DMSO) only.

### 2.6 RNA extraction and quantitative real‐time PCR (RT‐qPCR)

Total RNA was extracted from MC3T3 cells and primary osteoblasts using a Qiagen RNeasy Mini kit (Qiagen, Manchester, UK) according to the manufacturer's recommendations. The RNA samples were reverse‐transcribed into cDNA using Superscript II reverse transcriptase (Invitrogen) according to the manufacturer's instructions. RT‐qPCR was carried out in a Stratagene Mx3000P cycler with each reaction containing 50 ng template cDNA, 250 nM forward and reverse primers (Supplementary Table S1), and PrecisionPlus Mastermix (Primer Design, Chandler's Ford, UK). The cycle threshold (Ct) values for the samples were normalized to that of *Atp5b* or *Gapdh* (Supplementary Table S1) and the relative expression was calculated using the 2ΔCt method (Livak & Schmittgen, 2001).

### 2.7 Western blotting

Cells were scraped in RIPA lysis buffer containing protease inhibitors (Roche, Germany), and protein concentrations were determined using the Bio‐Rad protein DC assay (Bio‐Rad, Hemel Hempstead, UK). Protein (8–15 µg) was separated using a 10% Bis‐Tris gel and then transferred to a nitrocellulose membrane and probed with appropriate primary antibody (Supplementary Table S2), and appropriate HRP‐linked secondary antibody (Supplementary Table S3). Immune complexes were visualized by chemiluminescence using an ECL detection kit and ECL film (GE Healthcare, Amersham, UK). HRP‐conjugated anti β‐actin antibody (1:70,000, Sigma, Dorset UK) was used as a loading control. Densitometry analysis of protein was performed using Image J (https://imagej.nih.gov/ij/) (Baldari, Ubertini, Garufi, D'orazi, & Bossi, 2015).

### 2.8 E11 immunofluorescence

MC3T3 cells were plated on cover slips at a density of 6.3 × 10^3^ cells/cm^2^ and following treatment with FGF‐2, were fixed with 4% paraformaldehyde (PFA) for 15 min, washed in PBS and incubated in blocking buffer (1× PBS, 5% normal donkey serum and 0.3% Triton X‐100) for 1 hr at room temperature (RT). E11 antibody (Supplementary Table S2) was added to each well (1:900 in 1× PBS, 0.3% Triton X‐100 and 1% BSA) overnight at 4°C. Control cells were incubated with an equivalent concentration of goat IgG (Supplementary Figure S1). Wells were subsequently incubated with AlexaFluor‐conjugated donkey anti‐goat secondary antibodies (Supplementary Table S3) in the dark for 2 hr at RT. Glass coverslips were then mounted onto slides using ProLong Gold antifade reagent with DAPI (Life Tech) for nuclei staining (Dobie, Macrae, Huesa, Van't Hof, & Ahmed, 2014). The slides were finally visualized using a Leica DMRB fluorescence microscope and images were taken with a Leica DFC300 digital color camera (Leica, Milton Keynes, UK).

### 2.9 Transfection of MC3T3 cells with E11 siRNA

E11 siRNA and scrambled siRNA stocks (Qiagen) were diluted to 10nM. MC3T3 cells were plated at 8 × 10^3^ cells/cm^2^ and maintained in reduced serum medium. Cells were transfected as per manufacturer's instructions with complexes of E11siRNA with HiPerFect (Qiagen), while control cells were transfected with either complexes of scrambled siRNA, with HiPerFect; or HiPerFect alone. After 24 hr incubation at 37°C/5%CO_2_, FGF‐2 (10 ng/ml) was added for a further 24 hr to the cells containing the siRNA/HiPerFect complexes or the HiPerFect alone.

### 2.10 Immunohistochemistry

The knee joints of 6‐week‐old male FGF‐2 KO and WT mice (Chong et al., 2013), were fixed in 4% PFA for 24 hr before decalcification in 10% ethylenediaminetetraacetic acid (EDTA) pH 7.4 for approximately 3 weeks at 4°C with regular changes. Tissues were dehydrated and embedded in paraffin wax, using standard procedures, after which they were sectioned at 6 µm. Sections were dewaxed in xylene, rehydrated, and incubated at 37°C for 30 min in 1 mg/ml trypsin for antigen demasking. Endogenous peroxidases were blocked by treatment with 3% H_2_O_2_ in methanol. E11 and sclerostin antibodies (Supplementary Table S2) were used with appropriate IgG controls and secondary antibodies (Supplementary Table S3). The Vectastain ABC universal kit (Vector Laboratories, Peterborough, UK) was used according to the manufacturer's instructions. The sections were dehydrated, counterstained with haematoxylin and mounted in DePeX. Images were captured with Nikon Eclipse Ni microscope (Nikon, UK), fitted with Zeiss Axiocam 105 color camera (Carl Zeiss). The number of positively stained E11 osteocytes within diaphyseal cortical bone were calculated as a percentage of total osteocytes present.

### 2.11 Phalloidin staining for cell culture

MC3T3 cells were seeded at 1 × 10^4^ cells/cm^2^ and when sub‐confluent they were treated with 10 ng/ml FGF‐2 or 0.1% BSA for control cultures. After 24 hr, the cells were fixed in 4% PFA, rinsed in PBS and permeabilized in 0.1% (w/v) triton X‐100 (Sigma) in PBS for 10 mins, and then rinsed in PBS. The cells were incubated in 200 μl of Alexa Fluor 488‐conjugated phalloidin (Life Technologies, OR) (5 μM in PBS with 2% BSA) in the dark at RT for 3 hr. The cells were imaged on a Zeiss Axiovert 25s inverted microscope and digital imaging system (Carl Zeiss Microscopy, LLC, Oberkochen, Germany).

### 2.12 Phalloidin staining for histological sections

Femurs were decalcified as described above and then cryoprotected in 30% sucrose (w/v) at 4°C for 48 hr. The femora were cut in the mediolateral plane in serial longitudinal 20 μm thick‐sections using a cryostat and thaw‐mounted on gelatin‐coated slides for processing. Slides were dried at room temperature for 45 min, washed in PBS twice for 5 min each, and incubated with 0.1% Triton‐X 100 (Sigma‐Aldrich) for 30 min and then rinsed with PBS. Slides were then incubated with Alexa Fluor 488‐conjugated phalloidin (1:20; Thermo Fisher Scientific) for 1 hr. Bone sections were washed in PBS and mounted in VectaShield (Vector Laboratories). Preparations were allowed to dry at room temperature for 12 hr. Sections were imaged on a Zeiss LSM 710 Laser Scanning Confocal Microscope with 488 nm laser excitation and detection settings from 493 to 634 nm. Z‐stacks were produced with optimal Nyquist overlap settings using a 63×/1.4na oil immersion lens. Voxel sizes were 0.1 × 0.1 × 1.00 μm in *x*,*y*,*z* planes, respectively. A comparable region of interest was analyzed for osteocyte parameters in all samples located in the diaphyseal cortical bone. Image stacks were imported into Bitplane Imaris 8.2.0 software and algorithms were created with Imaris FilamentTracer to render and measure dendritic processes. Surface rendering was used for osteocyte cell body measurements.

### 2.13 Statistical analysis

Data are expressed as the mean ± standard error of the mean (S.E.M) of at least three replicates per experiment. Statistical analysis was performed by Student's *t*‐test, one‐way analysis of variance (ANOVA) or a suitable non‐parametric test. *p* < 0.05 was considered to be significant and noted as *p values of <0.01 and <0.001 were noted as ** and ***, respectively.

## 3 RESULTS

### 3.1 FGF‐2 promotes osteoblast E11 gene and protein expression

**Figure 1:**
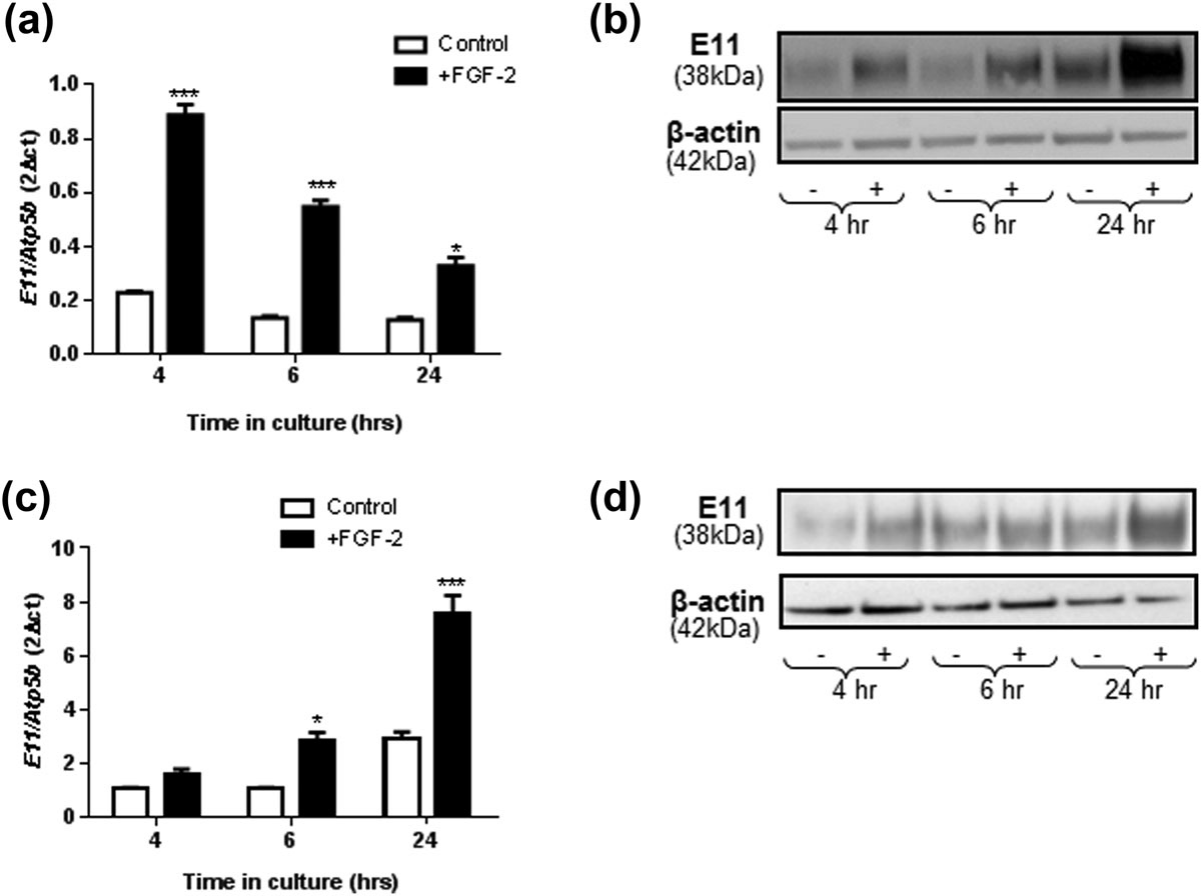
The effect of FGF‐2 (10 ng/ml) on (a) *E11* mRNA expression and (b) E11 protein expression in MC3T3 cells after 4, 6, and 24 hr challenge, where (+) is FGF‐2 treated cell, and (−) is untreated control. The effect of FGF‐2 (10 ng/ml) on (c) *E11* mRNA expression and (d) E11 protein expression in primary osteoblast cells after 4, 6, and 24 hr challenge, where (+) is FGF‐2 treated cell, and (−) is untreated control. Results were normalized to the *Atp5b* housekeeping gene and β‐actin for Western loading control. Data are presented as mean ± S.E.M for *n* = 3; *p < 0.05; ***p < 0.001 compared to untreated cells

Treatment of MC3T3 cells with 10 ng/ml FGF‐2 for 4, 6, and 24 hr stimulated *E11* mRNA expression in comparison to control cultures, at all time‐points examined (*p* < 0.05, Figure 1a). We observed a concomitant increase in E11 protein expression in these cells (Figure 1b). Stimulation of *E11* mRNA (*p* < 0.05, Figure 1c) and E11 protein (Figure 1d) expression by FGF‐2 was similarly noted in primary osteoblast cultures. The levels of FGF‐2 induced E11mRNA and protein were more prominent in the MC3T3 cells at the early time points (4 and 6 hr), whereas in primary cells these increases peaked at the later time points (24 hr) (Figure 1).

### 3.2 FGF‐2 promotes osteoblast–osteocyte differentiation

**Figure 2:**
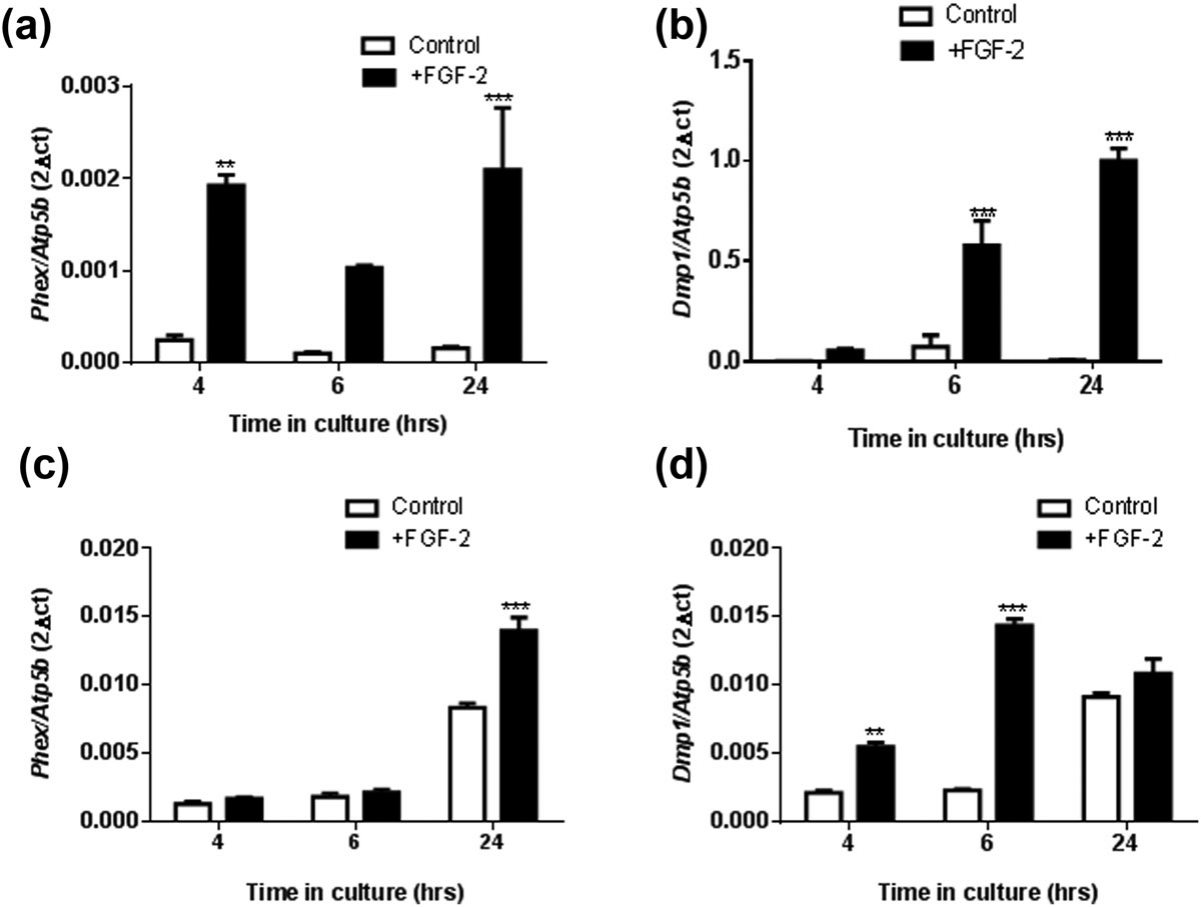
The effect of FGF‐2 (10 ng/ml) on the mRNA expression of (a) *Phex* and (b) *Dmp1* in MC3T3 cells after 4, 6, and 24 hr challenge. The effect of FGF‐2 (10 ng/ml) on the mRNA expression of (c) *Phex* and (d) *Dmp1* in primary osteoblast cells after 4, 6, and 24 hr challenge. Results were normalized to the *Atp5b* housekeeping gene. Data are presented as mean ± S.E.M for *n* = 3; **p < 0.01; ***p < 0.001 compared to untreated cells

In light of the increased E11 expression by FGF‐2, we next examined the expression of known osteocyte and osteoblast marker genes to determine whether exposure of osteoblast‐like cells to FGF‐2 promoted osteocytic differentiation. In MC3T3 cells, FGF‐2 increased the mRNA expression of the osteocyte marker *Phex* (phosphate regulating endopeptidase homolog, X‐linked) at 4 (*p* < 0.01), 6 (not significant), and 24 (*p* < 0.001) hours (Figure 2a). Similarly, *Dmp1* (dentin matrix protein 1) expression was significantly increased at both 6 and 24 hr in MC3T3 cells (*p* < 0.001; Figure 2b). In primary osteoblasts, both *Phex* and *Dmp1* mRNA expressions were also increased by FGF‐2 treatment, although the temporal changes were slightly different to those observed in the MC3T3 cells. Specifically, the stimulation of *Phex* expression by FGF‐2 was greater at late time points whereas the up‐regulation of *Dmp1* was noted at earlier time points when compared with MC3T3 cells (Figures 2c and 2d).

**Figure 3:**
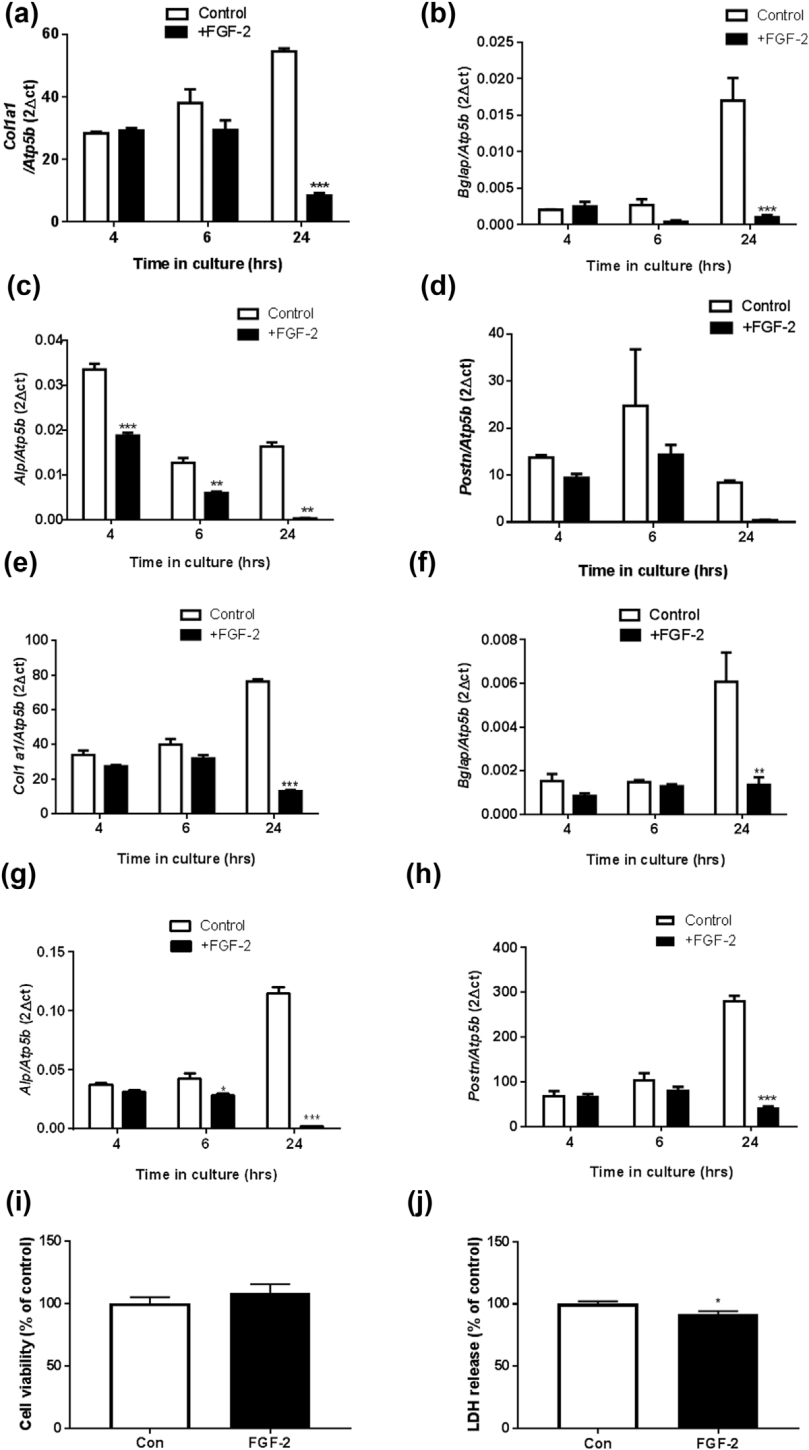
The effect of FGF‐2 (10 ng/ml) on the mRNA expression of (a) *Col1a1*, (b) *Bglap*, (c) *Alpl*, and (d) *Postn* in MC3T3 cells after 4, 6, and 24 hr challenge. The effect of FGF‐2 (10 ng/ml) on the mRNA expression of (e) *Col1a1*, (f) *Bglap*, (g) *Alpl*, and (h) *Postn* in primary osteoblast cells after 4, 6, and 24 hr challenge. Results were normalized to the *Atp5b* housekeeping gene. (i) Alamar blue assay for cell viability and (j) LDH release assay in FGF‐2 treated MC3T3 cells after 24 hr treatment. Data are presented as mean ± S.E.M for *n* = 3; *p < 0.05; **p < 0.01; ***p < 0.001 compared to untreated cells

In contrast to the increased expression of osteocyte markers by FGF‐2, there was a consistent downward trend in the mRNA expression of the osteoblast markers *Col1a1* (collagen type 1), *Bglap* (osteocalcin), *Alpl* (tissue non‐specific alkaline phosphatase), and *Postn* (periostin) in MC3T3 cells treated with exogenous FGF‐2 (Figure 3a–d). This down‐regulation of osteoblastic marker expression was most consistently observed 24 hr after exposure to FGF‐2, although *Alpl* expression was also reduced at 4 (*p* < 0.001) and 6 (*p* < 0.01), as well as 24 (*p* < 0.001) hour time points (Figure 3c). A similar down‐regulation of *Col1a1*, *Bglap*, *Alpl*, and *Postn* expression was also observed in FGF‐2 treated primary osteoblast cells, which was also most pronounced at longer (24 hr) times following FGF‐2 challenge (Figure 3e–h). Together these data indicate that exposure of MC3T3 as well as primary osteoblasts to exogenous FGF‐2 promotes early expression of both E11 and osteocyte markers, with a diminution in the expression levels of markers of the osteoblast phenotype following only at later time points.

Assessment of cell viability in the FGF‐2 treated MC3T3 cells by the alamar blue assay revealed that after 24 hr of FGF‐2 treatment there was no significant differences between the control and FGF‐2 treated cells (Figure 3i). We also observed a significant reduction in LDH release in our FGF‐2 treated cells (*p* < 0.05, Figure 3j) suggesting that there is less cell death. Taken together, these data are consistent with FGF‐2 promoting E11 expression and osteoblast–osteocyte differentiation in vitro.

### 3.3 FGF‐2 promotes E11 dependent osteocyte dendrite formation

**Figure 4:**
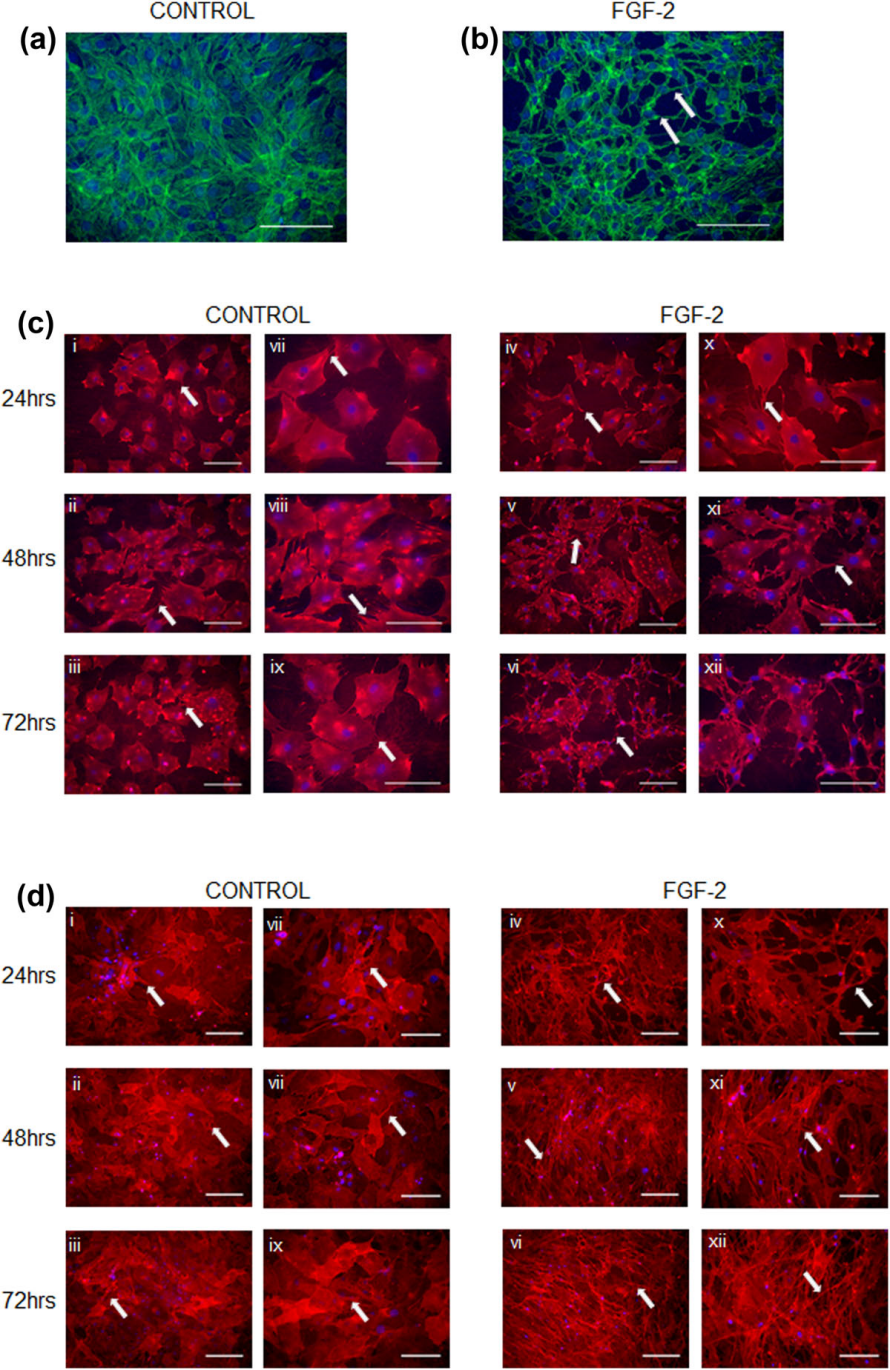
The effect of FGF‐2 (10 ng/ml) on MC3T3 osteoblast‐like cell morphology. (a) Phalloidin staining for F‐actin of control cultures, and (b) FGF‐2 treated cultures. Scale bar A & B = 150 μm). Immunofluorescence microscopy showing E11 expression and distribution in cells treated with FGF‐2 (10 ng/ml) for 24–72 hr in (c) MC3T3, and (d) primary osteoblasts. Note the arrows pointing at the dendrites. Images are representative of three separate experiments. Scale bar c & d (i–vi) = 200 μm; c & d (vii–xii) = 150 μm)

**Figure 5:**
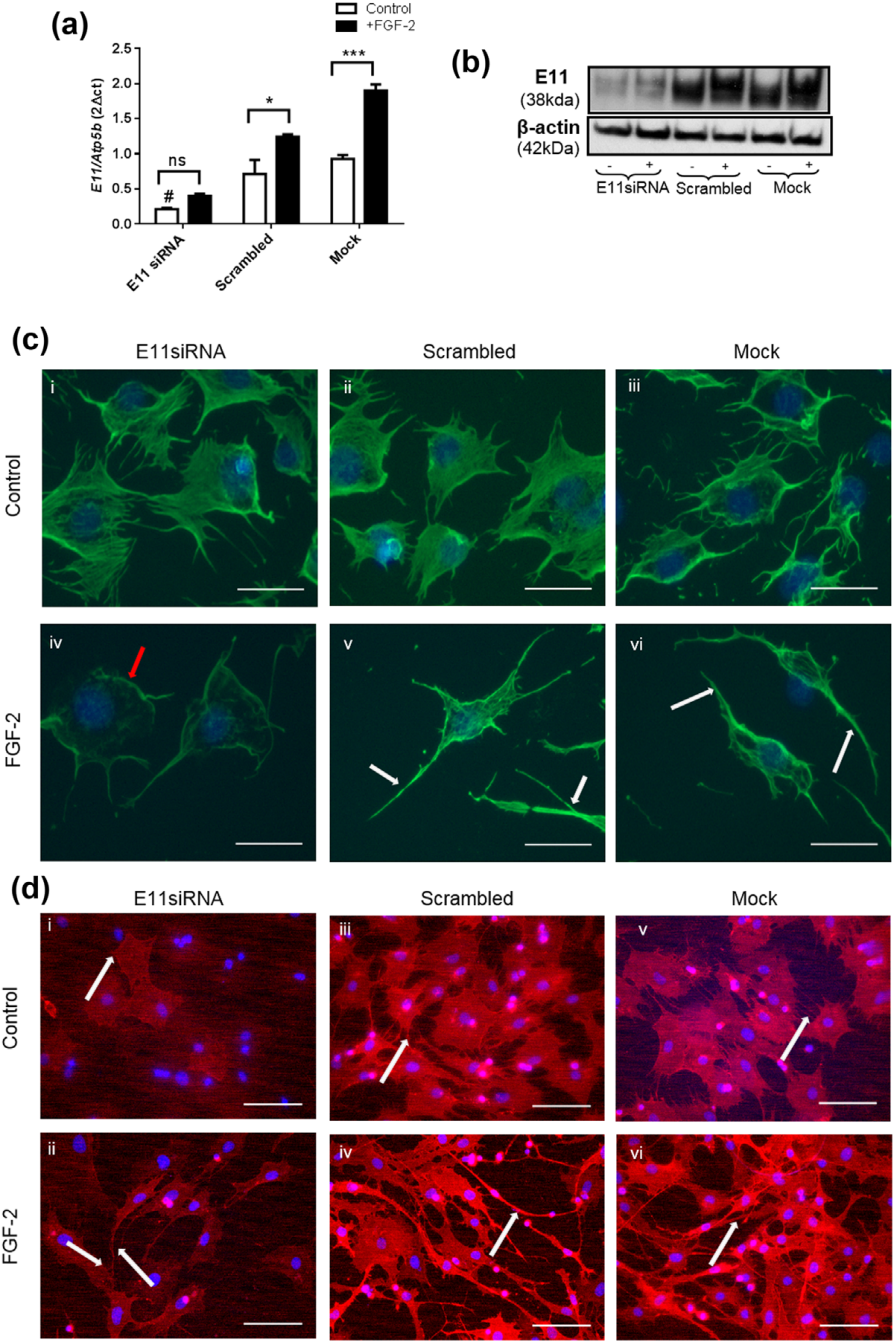
The effect of E11siRNA transfection on FGF‐2 (10 ng/ml) stimulation of *E11* (a) mRNA. Results were normalized to the *Atp5b* housekeeping gene. Data are presented as mean ± S.E.M for *n* = 3; *p < 0.05; ***p < 0.001 compared to untreated control cells; #*p* < 0.05 refers to significant decrease of E11siRNA control when compared to the controls of scrambled and Mock treated cells (b) The effect of FGF‐2 (10 ng/ml) on E11 protein expression after E11 siRNA transfection, where (+) is FGF‐2 treated cells, and (−) is untreated cells. Results are normalized to β‐actin for loading control. (c) Phalloidin staining for F‐actin in E11 siRNA, mock and scrambled cultures. Images are representative of three separate experiments. Scale bar = 100 μm. (d) Immunofluorescence staining for E11 localization in E11 siRNA, mock and scrambled cultures. Images are representative of three separate experiments. Scale bar = 150 μm

The differential regulation of osteoblast and osteocyte marker genes, including *E11*, by FGF‐2 strongly supports the tenet that FGF‐2 can induce osteocytogenesis. To examine this further, we next investigated whether FGF‐2 promotes the differentiation of MC3T3 osteoblast‐like cells into osteocytes with the adoption of their characteristic dendritic appearance through alterations to the intracellular cytoskeleton. We found that Phalloidin stained control cells displayed a typical rounded morphology with little evidence of dendrite formation (Figure 4a). In contrast, cells treated with FGF‐2 for 24 hr displayed numerous delicate dendrites radiating from individual cells and intertwining and connecting with dendrites from neighbouring cells, in a manner characteristic of an osteocyte‐like phenotype (Figure 4b). To clarify E11 involvement in this FGF‐2 induced change to dendritic phenotype, MC3T3 cells were challenged with FGF‐2 for 24–72 hr and immunostained for E11 (Figure 4c). All FGF‐2 treated MC3T3 cells exhibited modified morphology with numerous E11 positive dendritic processes radiating from the cell membrane (Figure 4c); these were only rarely observed in control cells. Furthermore, the distribution of intra‐cellular E11 expression changed with both time in culture and FGF‐2 treatment. In control cells, it was mostly uniformly distributed within the cytoplasm but after 72 hr in culture, cytoplasmic staining appeared less strong and the predominant staining was associated with focal accumulations at the cell membrane (Figure 4c). This redistribution of E11 to the cell membrane was more obvious and more rapid in the FGF‐2 treated cells, where it was achieved within only 24 hr of treatment (Figure 4c). Similarly, FGF‐2 promoted dendrite formation and the re‐distribution of E11 expression in primary osteoblast cultures (Figure 4d). To determine if the promotion of the osteocyte phenotype by FGF‐2 was E11 mediated we studied cells in which MC3T3 cells were transfected with E11 siRNA before being challenged with FGF‐2 for 24 hr. E11 gene (77% vs. mock control, 70% vs. scrambled control; *p* < 0.05; Figure 5a) and protein (Figure 5b) expression were silenced successfully by E11 siRNA transfection. Immunofluorescence labeling for E11 and phalloidin staining indicated that compared with mock or scrambled control cell cultures, cells treated with FGF‐2 developed less dendrites after silencing of E11 expression (Figures 5c and 5d).

### 3.4 FGF‐2 cell signaling in MC3T3 cells is mediated principally by phosphorylated ERK

**Figure 6:**
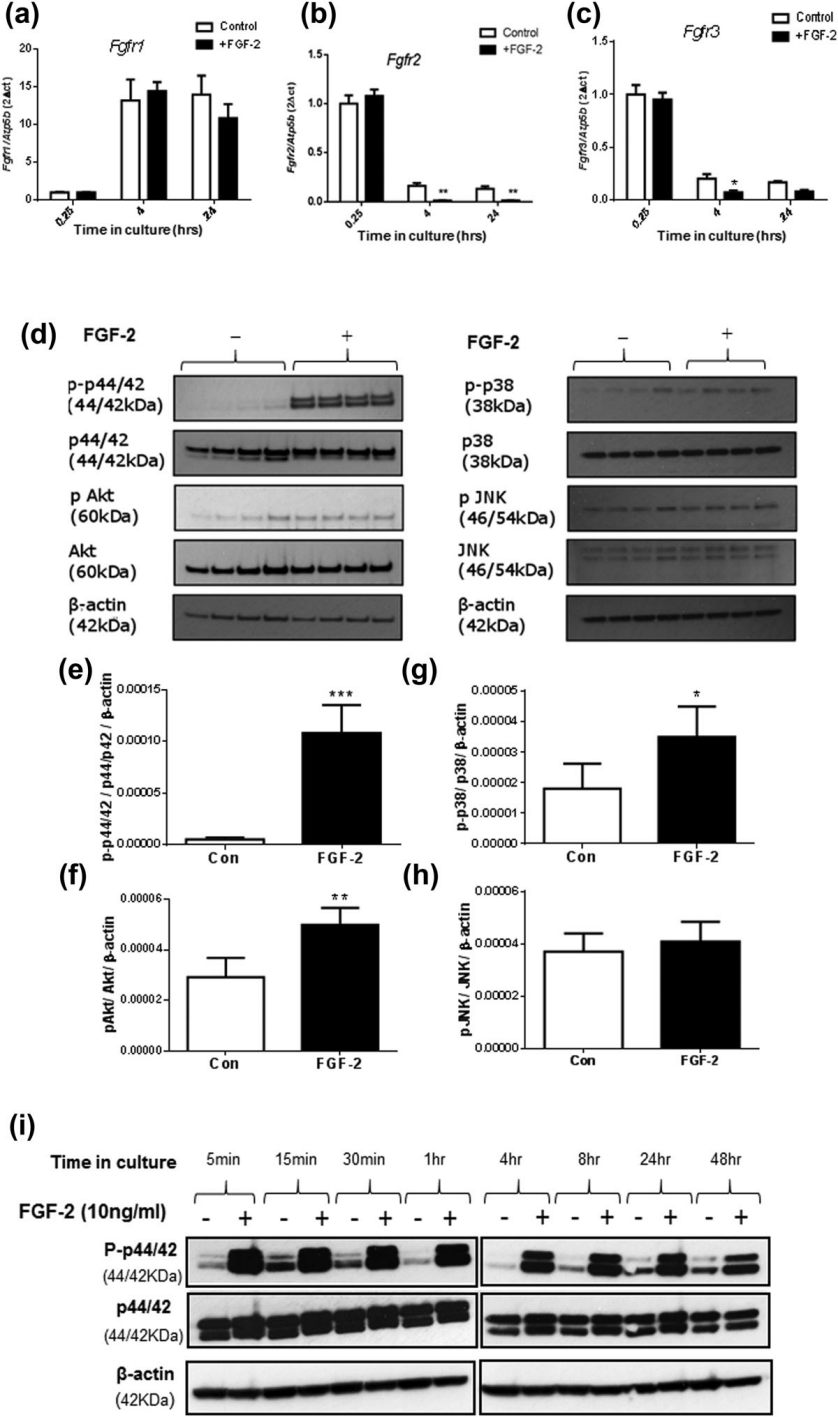
The effect of FGF‐2 (10 ng/ml) on the mRNA expression of (a) *Fgfr1*, (b) *Fgfr2*, and (c) *Fgfr3* in MC3T3 cells after 4, 6, and 24 hr challenge. Investigating the downstream signaling pathways involved in FGF‐2 stimulation of E11 expression. (d) Western blotting analysis of MC3T3 cells for phosphorylated and total p44/42 (ERK), Akt, p38, and JNK. Densitometry analysis of Western blotting revealed significant upregulation of activated (e) p44/42, (f) Akt, and (g) p38 in treated MC3T3 cells with FGF‐2 when compared to control cells. There was no significant increase in (h) JNK expression in both cultures. (i) Western blotting analysis of MC3T3 cells for phosphorylated and total p44/42, in MC3T3 cells treated with FGF‐2 when compared to control cells showed an increase in phosphorylated p44/42 in the treated cells at all time points. Results were normalized to the *Atp5b* housekeeping gene and β‐actin for Western blotting loading control. Data are presented as mean ± S.E.M for *n* = 4 and analyzed with student *t*‐test. *p < 0.05; **p < 0.01; ***p < 0.001

FGF receptors (*Fgfr*) 1, 2, and 3, but not *Fgfr4*, were found to be expressed by MC3T3 cells (data not shown). FGF‐2 treatment had no effect on *Fgfr1* expression at all‐time points studied (Figure 6a), however, it reduced *Fgfr2* (*p* < 0.01; Figure 6b) and *Fgfr3* (*p* < 0.05; Figure 6c) expression after 4 and 24 hr. Treatment of MC3T3 cells with FGF‐2 for 15 min revealed that of the pathways examined, there was particularly marked ERK (p44/p42) activation (*p* < 0.001; Figures 6d and 6e), while in comparison there was only slight activation of both Akt (*p* < 0.01; Figures 6d and 6f) and p38 (*p* < 0.05; Figures 6d and 6g), and no effect on JNK phosphorylation (Figures 6d and 6h). Furthermore, the temporal expression of ERK activation upon FGF‐2 treatment revealed a sustained activation over a 48 hr period (Figure 6i), which has been shown previously to be associated with pathways leading to cell differentiation (Pellegrino & Stork, 2006). These data suggest that ERK activation, rather than phosphorylation of alternative Akt, p38, or JNK mediated signaling pathways is likely most influential in regulating E11 downstream of FGF‐2.

**Figure 7:**
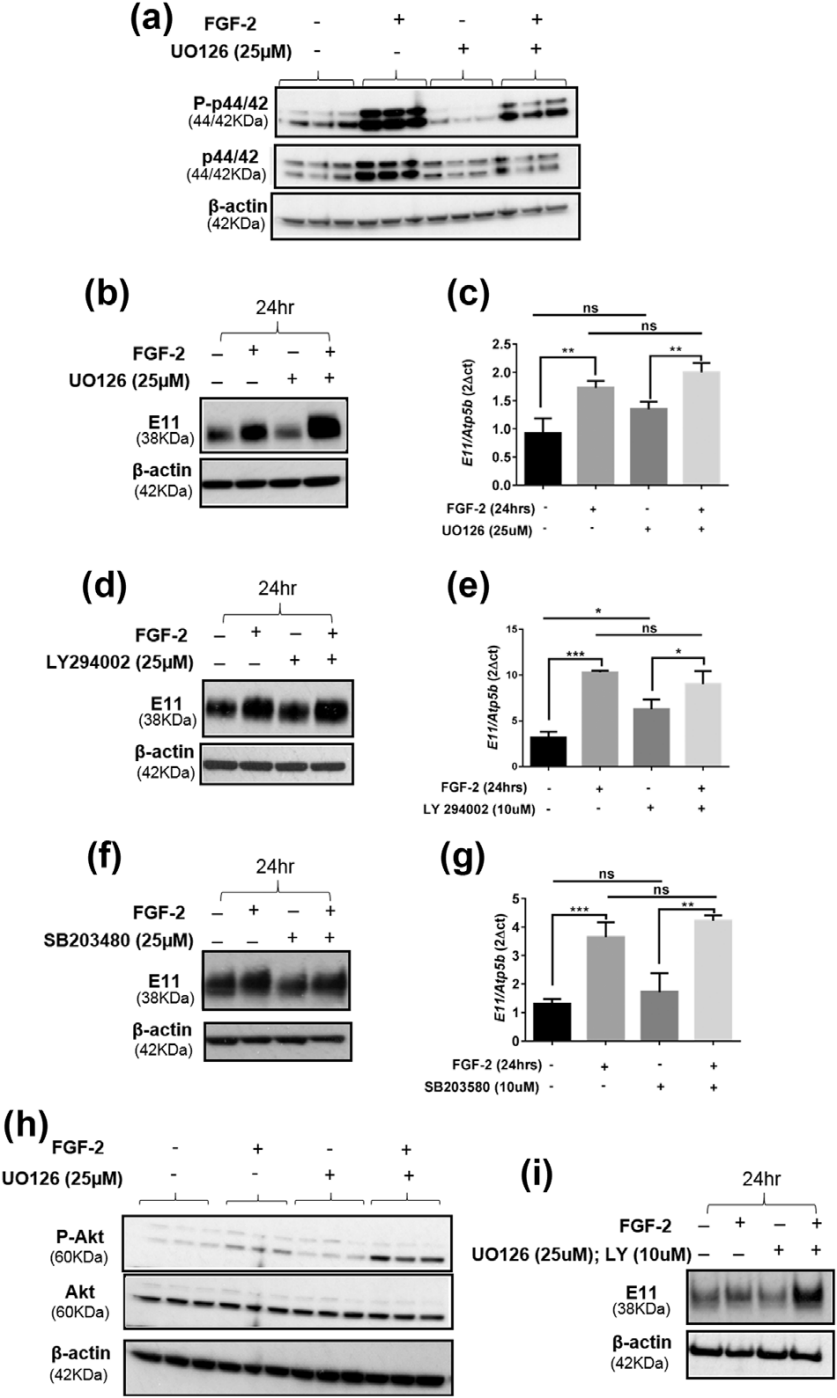
(a) Western blot analysis of ERK signaling in the presence (+) and absence (−) of U0126 (25 μm) incubation and subsequent FGF‐2 treatment. (b) Western blotting and (c) RT‐qPCR analysis of cells stimulated with FGF‐2 for 24 hr, in the presence or absence of U0126 (ERK inhibition). (d) Western blotting and (e) RT‐qPCR analysis of cells stimulated with FGF‐2 for 24 hr, in the presence or absence of LY294002 (Akt inhibition). (f) Western blotting and (g) RT‐qPCR analysis of cells stimulated with FGF‐2 for 24 hr, in the presence or absence of SB203480 (p38 inhibition). Effect of UO126 (25 μM) on Akt protein expression by (h) Western blotting. (i) Effect of U0126 (25 μM) and LY294002 (10 μM), P‐ERK, and P‐Akt inhibitors, respectively, on E11 protein expression. Results were normalized to the *Atp5b* housekeeping gene and β‐actin for Western blotting loading control. Data are represented as mean ± S.E.M for *n* = 3. Data are analyzed via one‐way ANOVA; *p* < 0.05 was considered to be significant. *p < 0.05

To further explore the likely role of MEK‐ERK signaling in FGF‐2 induced differentiation of osteoblast‐like cells into osteocytes, we next treated MC3T3 cells with the ERK inhibitor U0126 (25 μM) in the presence or absence of FGF‐2 (15 min). While ERK activation by FGF‐2 was blunted by U0126 (15 min) treatment (Figure 7a), the prolonged treatment of cells with U0126 (24 hr) did not affect the ability of FGF‐2 to enhance E11 gene expression (Figures 7b and 7c). Similarly, treatment of MC3T3 cells with p38 (SB203580) or PI3K (LY294002) inhibitors did not affect the ability of FGF‐2 to enhance E11 expression (Figure 7d–g). Further investigations indicated that Akt activation was increased in the presence of MEK inhibition by U0126 and FGF‐2 treatment (Figure 7h) and it is possible that this increased Akt signaling may be a compensatory change to allow FGF‐2 to promote E11 expression in the absence of full ERK activation (Figures 7b and 7c). However, the combined inhibition of MEK and PI3K signaling by the inhibitors U0126 and LY294002, respectively, did not affect the ability of FGF‐2 to enhance E11 protein expression (Figure 7i).

### 3.5 Deletion of FGF‐2 in vivo results in dysfunctional osteocytogenesis

**Figure 8:**
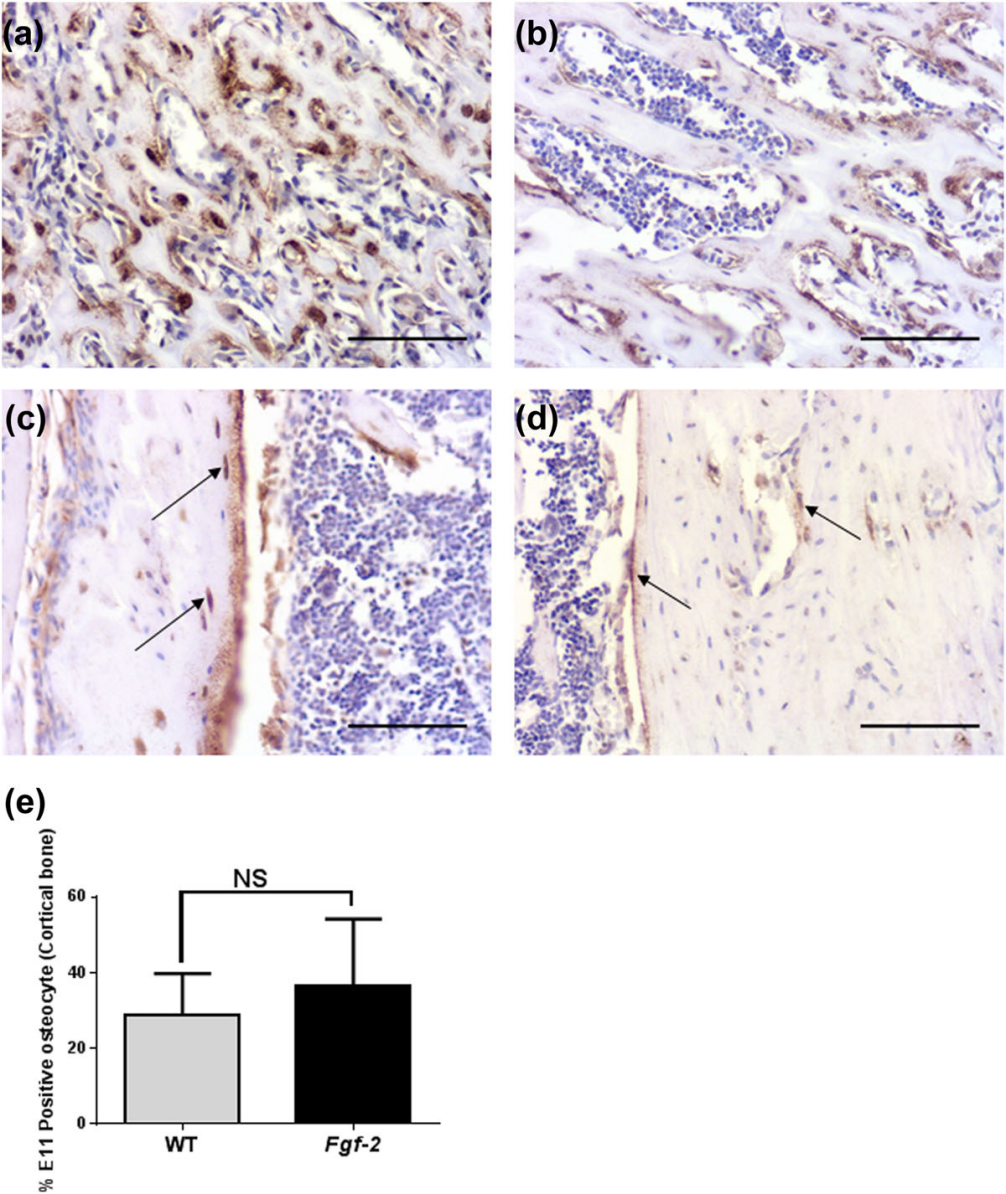
Sections of (a and b) trabecular bone and (c and d) cortical bone osteocytes from *Fgf‐2* KO and WT mice immunostained for E11. (a and b) Scale bar = 150 μm. (e) The number of E11 stained osteocytes was similar in cortical bone from *Fgf‐2* KO and WT mice. Images are representative of three mice

**Figure 9:**
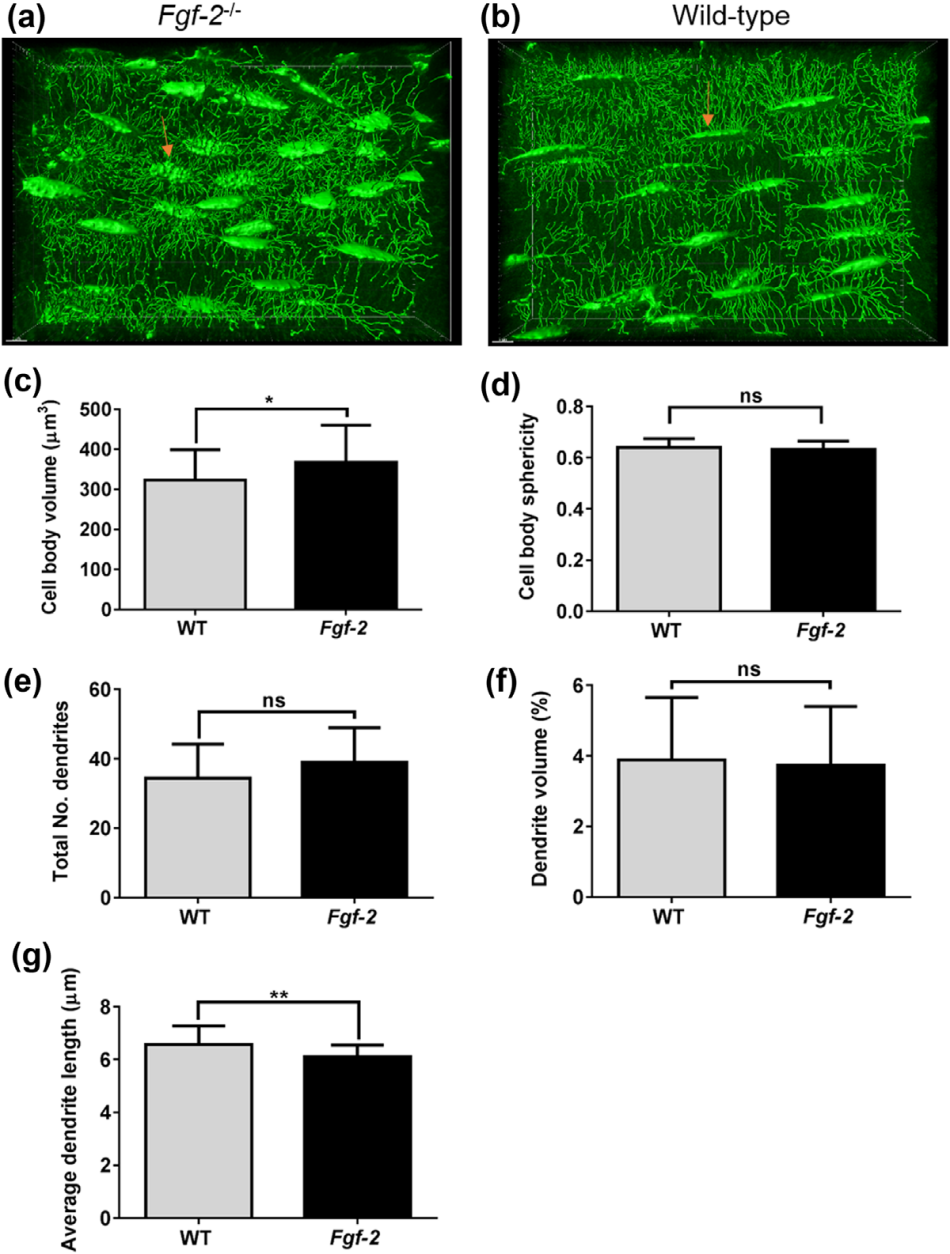
Phalloidin stained *Fgf‐2* KO and WT mice tibial cortical bone osteocytes and dendritic processes (arrow). Representative image of cortical bone osteocytes in both *Fgf‐2* KO (a) with larger cell body volume than the WT (b) as was confirmed by quantification (c), but no difference in cell spherical shape (d). While the total dendrite number (e) and volume (f), were not significantly different, the average length of the WT was longer than the *Fgf‐2* KO (g). Data are presented as mean ± S.E.M for *n* = 3 mice; *p < 0.05, **p < 0.01. Scale bar = 7 μm

Finally, we used immunohistochemistry to examine whether FGF‐2 KO mice exhibited altered skeletal E11 expression and distribution. Unexpectedly, E11 staining in osteocytes situated within trabecular and cortical bone of FGF‐2 KO mice appeared stronger than in osteocytes from WT bones (Figure 8a–d). Quantification of the number of E11 positive cells was, however, similar to those noted in bones from WT mice (Figure 8e). No differences in sclerostin expression or distribution in bones of FGF‐2 KO mice in comparison to those from WT mice were observed (data not shown). Histological analysis of osteocyte morphology in FGF‐2 KO mice revealed apparent increases in cell body volume (Figure 8a–d). To confirm and extend these results, we performed phalloidin staining of osteocytes in the cortical bone of FGF‐2 KO and WT mice (Figures 9a and 9b). We observed a significant increase in cell body volume (*p* < 0.05, Figure 9c) in concordance with our histological observations. Despite this, no differences in cell sphericity were observed (Figure 9d). Similarly, the total number of dendrites (Figure 9e) and the dendrite volume (Figure 9f) were unchanged between FGF‐2 KO and WT mice. We did, however, observe a significant decrease in average dendrite volume in FGF‐2 KO in comparison to WT mice (*p* < 0.01; Figure 9g), suggestive of dysfunctional osteocytogenesis in FGF‐2 KO mice.

## 4 DISCUSSION

The transmembrane glycoprotein E11, has recently been recognized to be an early driver of the osteoblast to osteocyte transition and the acquisition of the dendritic phenotype (Gupta et al., 2010; Zhang et al., 2006). Consistent with previous data, here we reveal that FGF‐2 is able to increase E11 expression and promotes osteocyte dendrite formation, likely independent of intracellular signaling pathways that may involve concomitant FGF‐2 induced ERK activation.

Previous brief reports have shown that FGF‐2 treatment of osteoblast‐like cells induces an increase in E11 expression and the appearance of the osteocyte phenotype (Gupta et al., 2010; Miyagawa et al., 2014). In this present study, we confirm and extend these observations in both MC3T3 osteoblast‐like cells and primary osteoblasts. The significant upregulation of *E11*, *Phex*, and *Dmp1* and down‐regulation of *Col1a1*, *Bglap*, *Alpl*, and *Postn* in the FGF‐2 treated cultures suggests that FGF‐2 promotes the differentiation of the osteoblast to the osteocyte stage. Concomitant with this, fluorescence microscopy of cultured cells also disclosed altered E11 expression and localization within the differentiating osteoblast in response to FGF‐2. The presence of increased E11 in the cytoplasm and perinuclear area suggests that FGF‐2 not only stimulates E11 expression, but also facilitates the translocation of E11 toward the cell membrane. Indeed, the ability of FGF‐2 to alter subcellular protein distribution is supported by a previous finding on the expression of Twist and Spry4 proteins in mesenchymal stem cells (Lai, Krishnappa, & Phinney, 2011). Here we observed E11 localization concentrated at the base of the dendritic spikes of the osteocytes after 24–72 hr of FGF‐2 treatment. E11 immunofluorescence localization at osteocyte dendritic projections has been reported in MLO‐Y4 osteocyte‐like cells and primary osteocytes isolated from long bones (Stern et al., 2012). It is, therefore, likely that this redistribution of E11 within the cell is necessary for the transformation of the osteoblast from a cuboidal shape to the osteocytic phenotype characterized by stellate‐like morphology with long dendritic processes (Zhang et al., 2006). We also reveal that these morphological changes do not occur because of altered cell proliferation, nor do they precede cell death, therefore, highlighting the role for FGF‐2 in regulating E11 expression and osteocyte differentiation in vitro.

The intracellular effects of FGF‐2 are activated via binding to its cell surface receptors, for example, FGFRs which have intrinsic receptor tyrosine kinase activity. Signaling pathways downstream of FGF‐2‐receptor binding are known to include ERK, p38, Akt, and PKC (Turner & Grose, 2010). Of those examined in the present study, ERK showed the most robust activation in response to FGF‐2 in MC3T3 osteoblast‐like cells; although p38 and Akt phosphorylation was also significant. Phosphorylation of ERK has been shown to mediate cell proliferation, differentiation, and matrix mineralization in human osteoblasts (Lai et al., 2001; Marie, Miraoui, & Severe, 2012). The sustained activation of the MEK‐ERK pathway and phosphorylation of ERK over long time periods suggests a central role for FGF‐2 stimulation of cell differentiation (Murphy, Mackeigan, & Blenis, 2003; Pellegrino & Stork, 2006). This is supported by studies that report the importance of ERK signaling in osteoblast initiation and commitment to the differentiation process (Lai et al., 2001), and in osteocyte dendrite formation (Kyono, Avishai, Ouyang, Landreth, & Murakami, 2012). Indeed, the conditional deletion of ERK ablates the formation of osteocytes with characteristic dendritic processes in vivo (Kyono et al., 2012).

Somewhat surprisingly, however, the MEK inhibitor, UO126 was unable to block FGF‐2's ability to promote E11 protein expression despite a significant reduction in ERK activation. Similar results were observed upon inhibition of PI3K/Akt and p38 signaling. These results suggest that alternative pathways may exist by which FGF‐2 is able to enhance E11 expression and osteocyte formation. Such pathways may include the activation of p38 and Akt. Previous reports have indicated that activation of p38 is involved in osteoblast differentiation (Hu, Chan, Wang, & Li, 2003) whereas Akt phosphorylation is associated with cell survival (Debiais et al., 2004). The down regulation of Akt by FGF‐2 has, however, also been reported in human and mouse cells (Chaudhary & Hruska, 2001). In our hands, however, the dual inhibition of Akt and ERK activation by LY294002 and U0126, respectively, did not result in a block in E11 expression by FGF‐2 and further work is required to unravel the signaling pathways that mediate FGF‐2 effect on the up‐regulation of E11 expression. The lack of JNK activation by FGF‐2 in this study is consistent with JNK phosphorylation (P‐JNK) mediating late osteoblast maturation (Matsuguchi et al., 2009).

Having shown that FGF‐2 promotes E11 expression in MC3T3 osteoblast like‐cells and murine primary osteoblasts, it was surprising to note that E11 protein expression by early osteocytes appeared to be increased in sections of bone from *Fgf‐2‐*deficient mice albeit no differences were noted in the number of E11 stained osteocytes. It is recognized that heparin‐like glycosaminoglycans can regulate the signaling behavior of FGF‐2 and, therefore, it is a possibility that in our cell culture experiments FGF‐2 is more available to the cells due to a less mature extracellular matrix being formed (Padera, Venkataraman, Berry, Godavarti, & Sasisekharan, 1999). Alternatively, the increased E11 staining intensity in the osteocytes from *Fgf‐2‐*deficient mice is maybe a compensatory response in an attempt to overcome the deficit in FGF‐2 related promotion of the osteoblast to osteocyte transition, potentially through the upregulation of other members of the FGF family. Similarly, it may simply be a consequence of the significantly increased cell body volume observed in FGF‐2 KO osteocytes. Indeed FGF‐2 has been reported to decrease chondrocyte hypertrophy in a murine metatarsal organ culture model and as such, may play a similar role in the formation of the osteocyte (Mancilla, De Luca, Uyeda, Czerwiec, & Baron, 1998). Our phalloidin staining also revealed a significant decrease in average dendrite length in FGF‐2 KO mice compared to WT mice; a similar phenotype to that observed in our bone specific E11 conditional knockout mice (Staines et al., 2017). This, therefore, suggests that the absence of FGF‐2 in vivo results in dysfunctional osteocytogenesis.

In conclusion, these data taken together show that FGF‐2 promotes the osteocyte phenotype and that this is mediated by increased E11 expression which is redistributed within the differentiating osteoblast. If further studies confirm this regulatory role for FGF‐2 in osteocyte formation, we will be in a better position to understand the full repertoire of FGF‐2 on bone cell function which may provide insights into the etiology of skeletal disorders such as osteoporosis and osteoarthritis.

## Supporting information

Additional Supporting Information may be found online in the supporting information tab for this article.

**Figure S1**. Immunofluorescence microscopy showing goat IgG control in MC3T3 cells counterstained with DAPI
**Table S1**. Sequences and source of primers used
**Table S2**. Primary antibodies used
**Table S3**. Secondary antibodies used
