# Supplementary material for: FGF‐2 promotes osteocyte differentiation through increased E11/podoplanin expression
Source: J Cell Physiol. 2018 Jan 23;233(7):5334–47. doi: 10.1002/jcp.26345 (PMC5900964; doi:10.1002/jcp.26345)
Supplement: Supplementary file 1 — Figure S1. Immunofluorescence microscopy showing goat IgG control in MC3T3 cells counterstained with DAPI Table S1. Sequences and source of primers used Table S2. Primary antibodies used Table S3. Secondary antibodies used [file JCP-233-5334-s001.docx]

**Supplementary Figure 1.** Immunofluorescence microscopy showing goat IgG control in MC3T3 cells counterstained with DAPI. Images are representative of three separate experiments. Scale bar = 200µm.

**
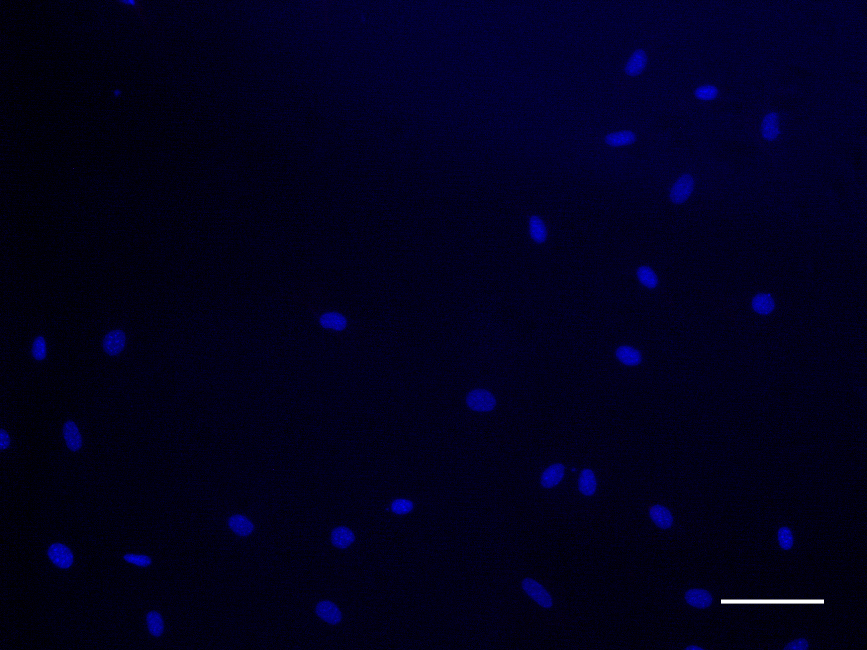
**

**Supplementary Table 1:** Sequences and source of primers used

| Gene | Source | S | Sequence (5’-3’) |
| --- | --- | --- | --- |
| *E11* | Primer Design | F | AACAAGTCACCCCAATAGAGATAAT |
|  |  | R | CTAACAAGACGCCAACTATGATTC |
| *Dmp1* | Primer Design | F | ATACCACAATACTGAATCTGAAAGC |
|  |  | R | CACTATTTGCCTGTCCCTCTG |
| *Phex* | Primer Design | F | CTAACCACCCACTCCCACTT |
|  |  | R | CCAATAGACTCCAAACCTGAAGA |
| *Sost* | Primer Design | F | TGAGAACAACCAGACCATGAAC |
|  |  | R | TCAGGAAGCGGGTGTAGTG |
| *Col1a1* | Primer Design | F | GCTCCTCTTAGGGGCCACT |
|  |  | R | CCACGTCTCACCATTGGGG |
| *Postn* | Primer Design | F | TTCCTCTCCTGCCCTTATATGC |
|  |  | R | CCTGATCCCGACCCCTGAT |
| *Bglap* | Eurofins MWG | F | TGCACGAAAGCAAGATGCTG |
|  |  | R | GGAGCGTCTGAATAGTCGCC |
| *Alpl* | Sigma | F | GGGACGAATCTCAGGGTACA |
|  |  | R | AGTAACTGGGGTCTCTCTC |
| *Fgfr1* | Qiagen | F | Not disclosed |
|  |  | R | Not disclosed |
| *Fgfr2* | Sigma | F | CCTGCGGAGACAGGTAACAG |
|  |  | R | CGCGTTGTTATCCTCACCA |
| *Fgfr3* | Qiagen | F | Not disclosed |
|  |  | R | Not disclosed |
| *Atp5b* | Primer Design | F | Not disclosed |
|  |  | R | Not disclosed |

**Supplementary Table 2.** Primary antibodies used

| **Antibody** | **Species** | **Source** | **Use** | **Dilution** |
| --- | --- | --- | --- | --- |
| E11 | Goat | R&D | Western Blotting | 1:1000 |
| Sclersotin | Goat | R&D | Western Blotting | 1:500 |
| p-p44/42 | Rabbit | Cell Signaling | Western Blotting | 1:1000 |
| Total p44/42 | Rabbit | Cell Signaling | Western Blotting | 1:1000 |
| pAKT | Rabbit | Cell Signaling | Western Blotting | 1:1000 |
| Total AKT | Rabbit | Cell Signaling | Western Blotting | 1:1000 |
| p-p38 | Rabbit | Cell Signaling | Western Blotting | 1:1000 |
| Total P38 | Rabbit | Cell Signaling | Western Blotting | 1:1000 |
| pJNK | Rabbit | Cell Signaling | Western Blotting | 1:1000 |
| Total JNK | Rabbit | Cell Signaling | Western Blotting | 1:1000 |
| β-actin (HRP-linked) | Mouse | Sigma | Western Blotting | 1:70000 |
| E11 | Goat | R&D | Immunohistochemistry | 1:500 |
| Sclerostin | Goat | R&D | Immunohistochemistry | 1:500 |
| E11 | Goat | R&D | Immunofluorescence | 1:900 |

**Supplementary Table 3.** Secondary antibodies used

| **Antibody** | **Source** | **Use** | **Dilution** |
| --- | --- | --- | --- |
| Rabbit anti-goat | Dako | Western Blotting | 1:3000 |
| Goat anti-rabbit | Dako | Western Blotting | 1:3000 |
| Donkey anti-goat | LifeTech | Immunofluorescence | 1:250 |
| Rabbit anti-goat | Vector Lab | Immunohistochemistry | 1:200 |
